# Research Orientation and Development of Social Psychology’s Concept of Justice in the Era of Cloud Computing

**DOI:** 10.3389/fpsyg.2022.902780

**Published:** 2022-05-25

**Authors:** Yongyan Zhao, Desheng Du

**Affiliations:** ^1^School of Humanities and Law, Harbin University, Harbin, China; ^2^School of Marxism, Yantai University, Yantai, China

**Keywords:** cloud computing, social psychology, justice concept, research orientation, Markov algorithm

## Abstract

With the maturity and rapid expansion of social psychology, great progress has been made in the integration of social psychology with other disciplines. From the very beginning, social psychology is destined to have a diversified and multidisciplinary research orientation and disciplinary nature, which also makes it difficult for social psychology to be defined in a single disciplinary field and a single research method. With the rapid development of the Internet, the emergence of cloud computing technology not only facilitates the orientation of psychological research, but also promotes the emergence and development of some new psychological disciplines. Therefore, the purpose of this paper is to study the orientation of social psychology and its current development in the context of cloud computing era. This paper collects, organizes, and integrates the research data of college students’ view of justice from the perspective of social psychology through cloud computing technology, and uses empirical research methods to conduct in-depth research on people’s view of justice in social psychology. This paper collects the data reports of college students on social justice issues through cloud computing technology to make the results more accurate. The experimental results show that nearly 70% of college students pay more attention to social justice issues. This data clearly reflects the optimistic trend of people’s attention to justice issues in social psychology.

## Introduction

Social psychology has slowly developed into a new type of discipline in the last century. On the one hand, the innovative results produced by social psychology through the gradual integration with other disciplines of society are applied to every field of society; on the other hand, due to the limitations of social psychology itself, its development has stopped. As a technology emerging in recent years, cloud computing can collect and integrate a large amount of social psychology research data through its existing parallel computing and distributed computing technology. This not only speeds up the research progress of social psychology, but also saves a lot of time for people’s research on social psychology. And how to adapt to the development research of the social psychology orientation and status quo in the cloud computing era has become a practical problem that must be solved.

In the research orientation of social psychology, there are many scholars who have studied it and achieved good results. For example, Rubin (2017) proposed an exploratory measurement method (EMMM) based on multiple motivations in social psychology. This approach enables researchers to conduct rapid exploratory assessments of a relatively large number of general motivations, facilitating the study of the divergence and convergence effectiveness of their projects (Rubin, 2017). In India, social psychologists have attempted to instill the missing picture of an “indigenous perspective” from a cultural vantage point. Pitts (2019) proposed communication taste as a new dimension of social taste, making its research in the fields of language communication and social psychology both theoretical and practical. Kruglanski et al. (2017) suggests shifting attention to the theory, methods, and applications of social psychology, which could lift social psychology from its current slump and allow it to fulfill its potential as an indispensable social science. Banerjee et al. (2021) attributes causes to actions to make them logical and easier to understand, and in social psychology, attribution is the process by which individuals explain the causes of actions and events. Brannigan (2021) kept the research records of classic experiments in university archives, opening up a new research avenue for social psychology students. Watanabe and Laurent (2020) examined four methods that are frequently used in the study of religion in social psychology and proposes improvements on theoretical issues to promote a clearer understanding of how religion affects human behavior. Tolstykh (2020) discusses the turn to social psychology and discusses the trend toward the development of cultural history theory to social psychology. Carrer and Bruzzone (2017) research on information extraction algorithms in social psychology based on maximum entropy hidden Markov models. He combined Hidden Markov and Maximum Entropy to study the algorithm of information extraction in social psychology and analyzed the accuracy of information extraction in social psychology. Although their research has made great contributions to social psychology, there is still a large gap in the research orientation of social psychology. Although social psychology has been introduced in detail, there are still some problems in its development.

This paper studied the research orientation and status quo development of social psychology in the context of cloud computing era, which can serve as a reference for future research on social psychology in the context of cloud computing era. In this paper, the Markov algorithm is combined with cloud computing technology to build a psychological simulation model, so as to explore the changes of people’s dynamic psychological structure.

The innovations of this paper are as follows:

1. This paper used cloud computing technology to collect, organize and integrate data on the design ideas of the social psychology research on college students’ view of justice. And it adopted the method of empirical research to study the attitude of college students to the view of social justice, and then analyzes the problem of social justice, and draws a conclusion.
2. This paper uses cloud computing technology and the theoretical concepts of social psychology to analyze the attitude of college students to social justice issues and provides a research route for people to use cloud computing technology to study psychological issues in the future, which has certain research significance.
3. In the discussion of the cultivation path of college students’ view of justice, this paper proposes to draw lessons from social psychology theories, such as attribution theory, conformity theory, attitude change theory, and face theory, and the cultivation is more feasible and practical.

## Based on the Research Orientation and Related Methods of Social Psychology in the Era of Cloud Computing

**Figure 1:**
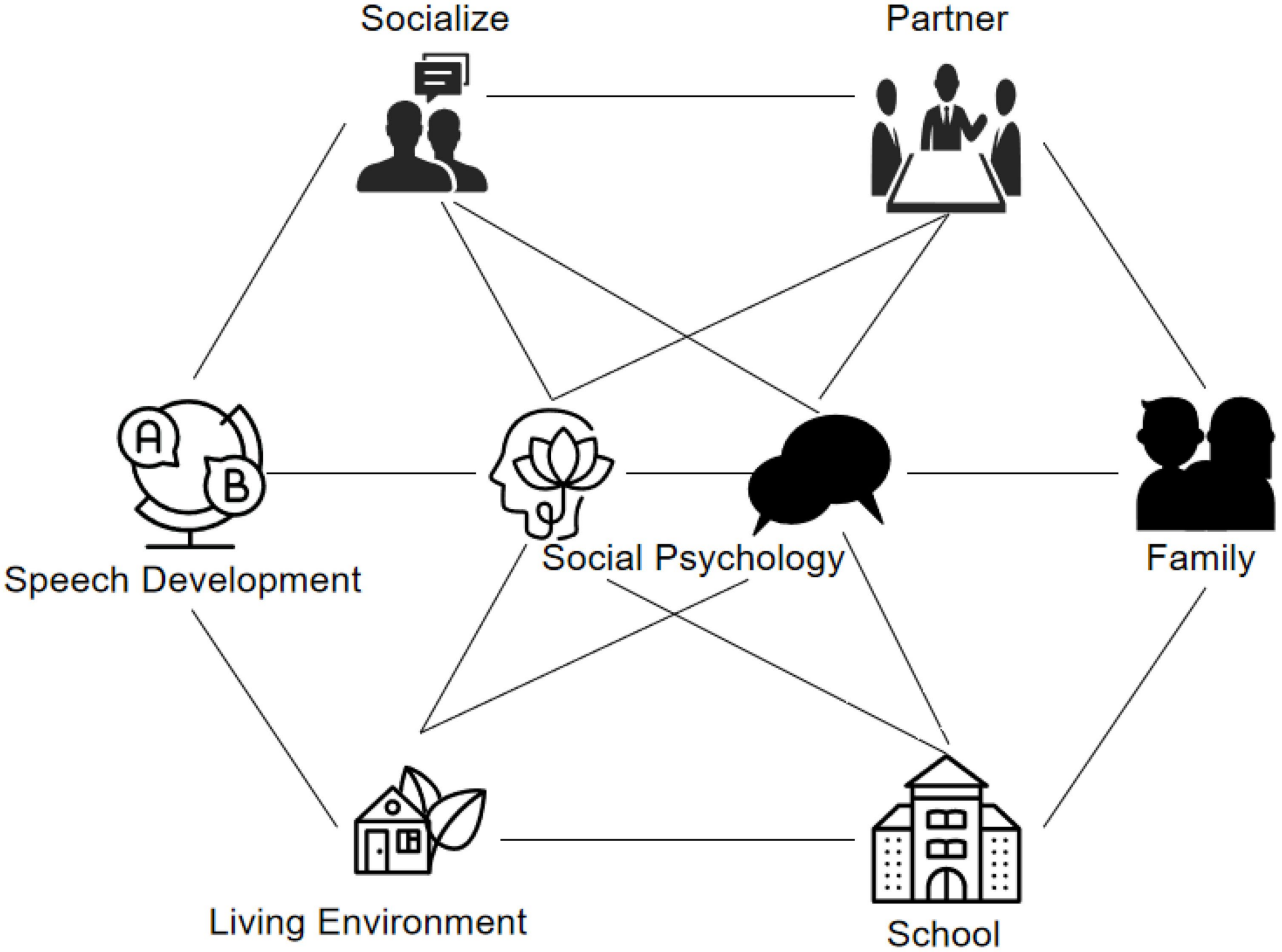
Social psychology individual level research areas.

Social psychology research based on cloud computing era refers to the theoretical analysis of interpersonal relationships at the individual level and social group level from the perspective of cloud computing. The individual-level research areas of social psychology are shown in Figure 1.

As shown in Figure 1, the content of research at the individual level based on social psychology in the cloud computing era includes: the process of individual socialization, interpersonal communication, partners and family members and living environment, and the impact of school on the individual’s psychology.

### Concepts Related to Social Psychology

Traditional social psychology still seems to be the social psychology of psychological orientation research, and traditional social psychology can also refer to the practitioners of psychology. But since people’s mental states are considered in different social contexts, there are many previous social psychological concepts that have changed over time (Meyerhuber, 2020). In terms of variables, although social sciences only integrate culture as a variable in their research, as a result, cross-cultural social psychology may objectively bring psychology-oriented social psychology closer to sociologically oriented social psychology. This variable does not belong to a constant variable. Its emergence may be that traditional social psychology is largely different from new social psychology (Lisitsyna, 2020; Tsuji, 2020).

### Status Quo of Cloud Computing Psychological Service Platform

**Figure 2:**
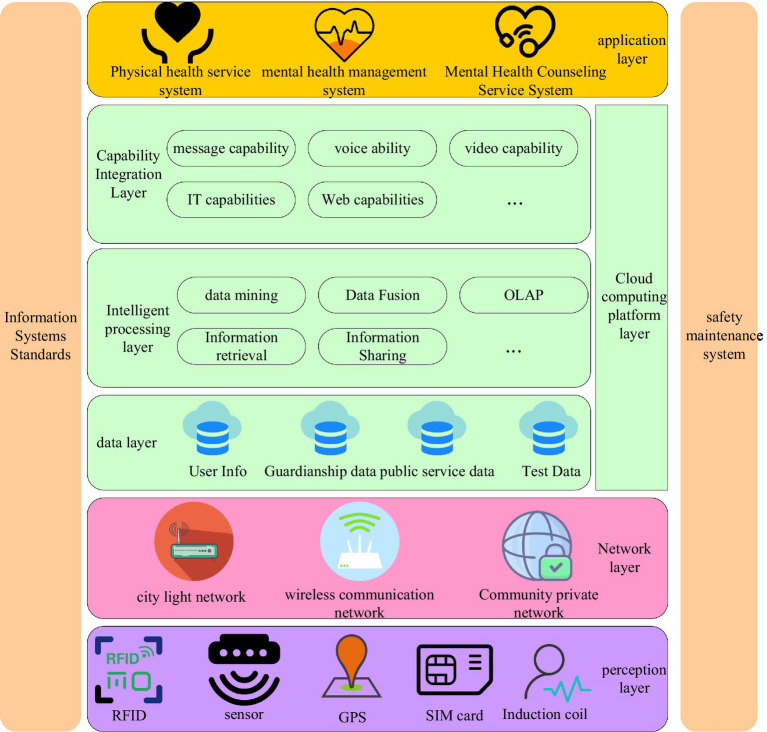
Cloud computing-based mental health service platform.

Cloud computing technology can provide an information collection platform for social psychology research orientation, so as to better provide data support for social psychology research orientation and its current development. For example, a cloud computing-based mental health service platform can collect customers’ psychological research data. And this cloud computing-based mental health service platform can collect various data sources collected by the terminal, and analyze and process various data sources through cloud computing technology. Some scholars also believe that taking mental health issues as a long-term research object, collecting users’ psychological characteristics data in real time like chronic diseases, and storing and subsequent classification of users’ specific psychological characteristics data, thus using the cloud computing-based mental health service platform as a long-term monitoring service. In this paper, by designing a large-scale cloud computing-based mental health data center analysis platform, this cloud computing-based mental health service platform can meet the research needs of professionals. The design of the mental health service platform based on cloud computing is shown in Figure 2.

As shown in Figure 2, the architecture mainly includes two parts: a mental health database and a smart service platform. The mental health management system is based on measuring the heartbeat, and manages different aspects of the user’s mental health from the aspects of instant messaging and personal center, and strives to solve psychological problems. This paper provides an opportunity for psychologists to express themselves and communicate with users, which is also the purpose of the cloud computing-based mental health service platform to achieve a win-win for all users and psychologists. In terms of social psychology research, cloud computing can bring the most accurate, effective, and complete scientific analysis data for social psychology research, which can promote the development of psychological research orientation and status quo research.

### Theoretical Analysis Tools Related to Evolutionary Algorithms

#### Markov Chain Modeling and Analysis

Markov chain is the main analytical tool for theoretical analysis of evolutionary algorithms. The evolution process of the evolutionary algorithm can be regarded as the transition of the population between different states. The evolutionary algorithm without adaptive strategy only relies on the current state and the transition matrix in the current state for each state transition. The Markov chain model can define the process of two-state changes in psychological research, combining the characteristics of cloud computing. In this paper, a simulated mental model is built with cloud computing technology, and concepts, such as mental energy, mental strength, and mental entropy, are given to describe the psychological characteristics and states (Alyousifi et al., 2021). Among them, the Markov chain L is defined, and the transition matrix of the homogeneous Markov connection is *P*, then the transition matrix *P* is as follows:


(1) $$P\left[\begin{array}{cc} I & 0\\ Z & T \end{array}\right]$$

(2) $$m=1{\left(I-T\right)}^{-1}$$

Among them, the matrices *Z* and *T* are identity matrices of the same order, and 1 is a vector (1…1). Based on this formula, the expectation of the first arrival time of the evolutionary algorithm can be analyzed. Based on the first arrival time, the conditional expectation of the first arrival time can be given (Ma et al., 2019; Smith et al., 2019). Among them, the identity sub-matrix I is the probability transition matrix of L on the absorbing state; R is the transition probability of each state to each absorbing state in the target state; T is the transition matrix between each state in the state subset. According to the theorem, the first arrival time of the Markov chain can be obtained as follows:


(3) $$F\left[\tau |L_{0}=X\right]=\overset{+\infty }{\underset{K=0}{\sum }}kP\left(\tau =k|L_{0}=X\right)$$

#### Transfer Analysis

Evolutionary Algorithms Time Complexity Analysis Tool “Transition Analysis.” The transition analysis first defines the distance function between the population individuals and the optimal solution. It estimates the average first reaching time of the algorithm by estimating the average distance that the algorithm advances to the optimal solution in each generation, and then further analyzes the upper and lower bounds of the average time complexity of the evolutionary algorithm. This method is the mainstream method for time complexity analysis of evolutionary algorithms.

For a population’s optimization process fitness value sequence {X_0_,…,X_t-1_}, its speed (Drift) ∆_t_(X) can be defined as:


(4) $${△}_{t}\left(X\right)=E\left[D\left(X_{t}\right)-D\left(X_{t+1}\right)|X_{0}\mathrm{|,}\ldots ,X_{t-1}\mathrm{,|}\right]$$

Among them, F is the complete set of the population. If O(X) ≥ 0 holds, then {$D{\left(X\right)}_{t}$,*t* = 0,1,…} is called an upper martingale (Long et al., 2019; Alyousifi et al., 2020). At this time, the first arrival time of the algorithm can be divided by the speed, such as the distance function, as follows:


(5) $$E\left(t\right)=\frac{D{\left(X\right)}_{t}}{\Delta_{t}\left(X\right)}$$

The upper and lower bounds of the average time complexity of evolutionary algorithms can be analyzed based on transition analysis. The upper bound theorem of transfer analysis is:


(6) $$F\left[D\left(\xi_{t}\right)-D\left(\xi_{t+1}\right)|\xi_{t}\right]\geq C_{t}>0$$

(7) $$F\left[\tau |\xi_{0}\right]\leq \frac{D\left(\xi_{0}\right)}{C_{t}}$$

Among them, the first arrival time can be given, and $\xi_{0}$ is the initial population of the evolutionary algorithm. The following theorem of transfer analysis is:


(8) $$F\left[D\left(\xi_{t}\right)-D\left(\xi_{t+1}\right)|\xi_{t}\right]\leq C_{u}$$

(9) $$F\left[\tau |\xi_{0}\right]\geq \frac{D\left(\xi_{0}\right)}{C_{u}}$$

Similarly, where the first arrival time can be given by Equation (9), $\xi_{0}$ is the initial population of the evolutionary algorithm (Leyva et al., 2019; Nasiri et al., 2019).


(10) $$\Delta =\frac{u_{n}+1-2u_{n}+u_{n}-1}{{\left(\Delta t\right)}^{2}}$$

Replacing $u_{n+1}$ and $u_{n}$ with the expected value has.


(11) $$\Delta =\frac{r_{k}\left(u_{n+1}-2u_{n}+u_{n-1}\right)+\left(1-r_{k}\right)\left(u_{n}-u_{n-1}\right)}{{\left(\Delta t\right)}^{2}}$$

Then convert the difference quotient on the right side of Equation (11) into the representation of the derivative, we have.


(12) $$\Delta =c_{k}\frac{d^{2}x}{dt^{2}}+\frac{1-c_{k}}{\Delta t}\frac{dx}{dt}$$

A new kinetic equation has been obtained.


(13) $$r_{k}\frac{d^{2}x}{dt^{2}}+\frac{1-r_{k}}{\Delta t}\frac{dx}{dt}+c_{k}x$$

It can be associated with the Markov chain length $L_{k}$ in the algorithm. When the number of cycles $L_{k}$ corresponds to the time unit τ, it can be substituted into Equation (13) to get.


(14) $$\frac{d^{2}x}{dt^{2}}+\lambda_{k}\frac{dx}{dt}+\frac{u_{k}}{r_{k}}x=0$$

In the equation, $\lambda =\frac{1-r_{k}}{\Delta t}\frac{L_{k}}{\tau }$ is the damping coefficient in elastic mechanics, and the greater the moving speed of the particle $\frac{dx}{dt}$, the greater the blocking force generated by the damping coefficient.

#### Analysis of the Local Convergence of the Algorithm of the Dynamic System Model

For the dynamic equation of, $z_{k}$ can be approximated as a constant, then this is a second-order linear ordinary differential equation, and it is easy to obtain the characteristic equation as.


(15) $$\lambda^{2}+z_{k}=0$$

The equation has an imaginary eigenvalues, which is $\lambda_{1,2}=\sqrt{z_{k}t}$, and i is an imaginary unit. At this time, the general solution of the equation can be solved as a simple harmonic vibration solution.


(16) $$x\left(t\right)=c_{1}\cos\left(\sqrt{z_{k}t}\right)+c_{2}\cos\left(\sqrt{z_{k}t}\right)$$

Among them, C1 and C2 are undetermined constants, which can be determined by the initial conditions. The mainstream developmental psychology model can be expressed as a function:


(17) $$z_{t}=f\left(a,b,c\ldots \right)$$

Among them, $z_{t}$ represents the state of the individual’s psychological structure or behavior pattern at time *t*, and *a*, *b*, and *c*, etc., represent $z_{t}$ variables that have an impact. The point of view of the dynamic system theory based on cloud computing can be expressed as:


(18) $$z_{t}=f\left(z_{t-1},a,b,c\ldots \right)$$

This means that an individual’s “present” behavior is not only influenced by factors, such as genes and environment, but also by an individual’s “past” behavior.

## Social Psychology Dynamic System and Experimental Investigation Research

### Experimental Investigation and Research Methods Based on Cloud Computing

#### Empirical Research Method

Based on cloud computing technology, this paper conducts statistical analysis on the research data of college students’ psychology by collecting cloud databases of major schools. These data are obtained by random sampling surveys and interviews of students through interactive questionnaires in major schools. This paper uses cloud computing technology to analyze the results of these questionnaires and the data labels generated by the interviews. It finds out the social psychological problems and analyzes the reasons.

#### Comparative Research Method

This paper makes full use of the current research materials of social psychology, compares and analyzes the characteristics of many social psychology concepts, combines their ideas and methods, learns from each other’s strengths, brainstorms, and finally improves my research conclusion. The application of cloud computing to psychological research in this paper can provide a rich sample for social psychology research. The application of cloud computing means to conduct social psychology research can obtain more real, accurate, and timely big data samples and can provide a full-data model research method for social psychology research orientation and status quo development.

#### Research Methods of Multidisciplinary Integration

Psychology and political science are based on many disciplines, such as political science, sociology, psychology, and education. In view of this characteristic, this paper makes full use of related ideas from philosophy, political science, and sociology in the research process.

### Dynamically Changing Psychological Structure

**Figure 3:**
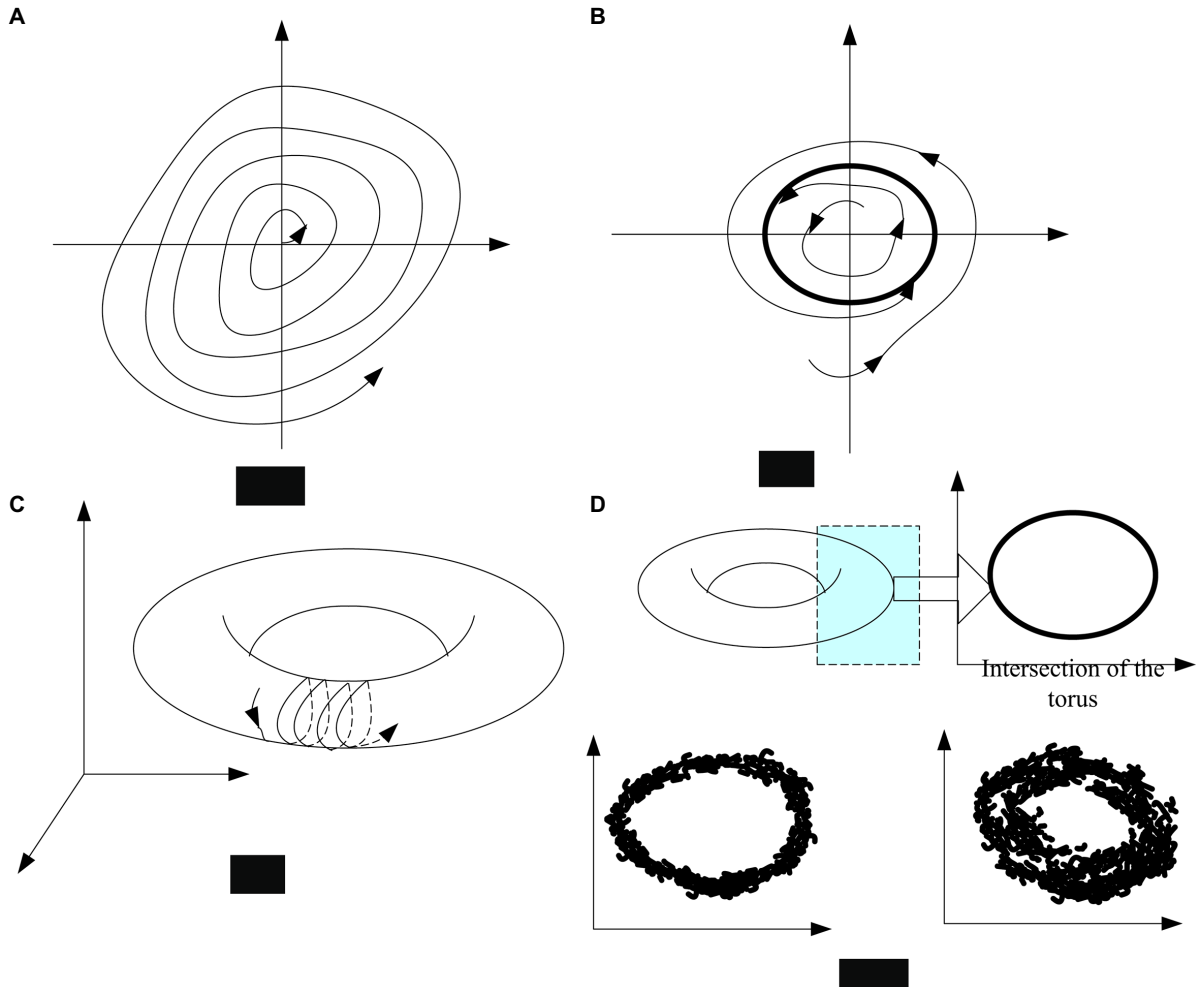
Four types of attractors. **(A)** Fixed-point attractor; **(B)** periodic attractor; **(C)** torus attractor; and **(D)** chaotic attractor.

Repeating the same behavior over and over a period of time results in a fixed pattern of behavior, which will mean that people stay in a state of mind that is stable. For example, spending time with people who are high in happiness can keep a person emotionally stable for a period of time. Of course, this change may be related to the formation and loss of natural connections or circuits in the cerebral cortex. Likewise, loss of attractiveness means disruption of cognitive and behavioral systems. To sum up, the types of attractors that are ubiquitous are shown in Figure 3.

As shown in Figure 3, as a dynamic system based on psychology, the changes of this psychological dynamic system at the state level can be described. Among them, the relatively stable psychological structure is the attractor, and the volatile and elusive psychological structure can be called the repulsor. Compared with the traditional description of the psychological structure, this way of expression can better reveal the changing nature of the psychological structure. It can be seen from the description of people: the traditional psychological description can be a static description without changing characteristics.

### Multi-Level Nested Mental Structure

**Figure 4:**
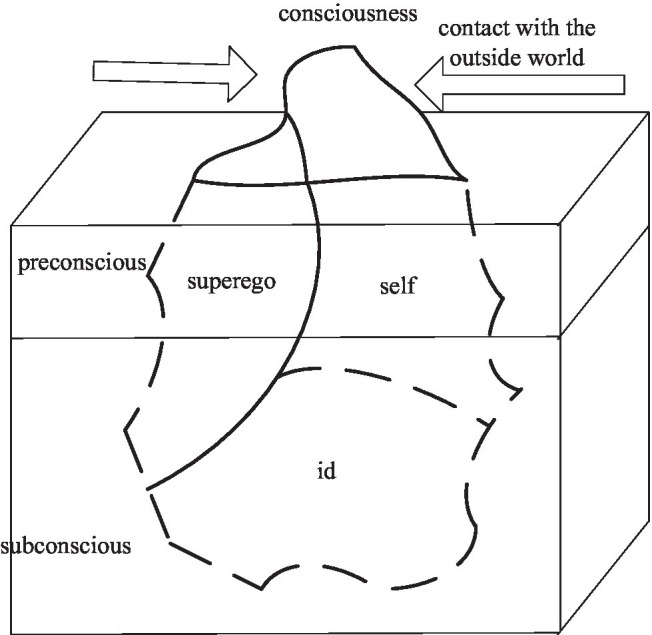
Mental structure model.

A strong system structure means that a scientific system cannot be seen as an individual in an individual world isolated from the outside world. It is a complex system with multiple stages depending on the specific nervous system, body, and environment. One is in the brain, while the brain is in the body, and the body is surrounded by its surroundings. Its multi-level nested psychological structure is shown in Figure 4.

As shown in Figure 4, in a given body and state, these constituent mental structures continuously work together to form a whole. Therefore, people’s environment and themselves also belong to a part of the “psychological structure” and cannot be separated from it alone. The narrow dynamic system orientation is more radical. It even believes that abstract organizations, such as mental structures, do not actually exist, and only real-time dynamic processes are the only real ones (Ardianto and Chhetri, 2019; Ben-Ammar et al., 2019).

**Figure 5:**
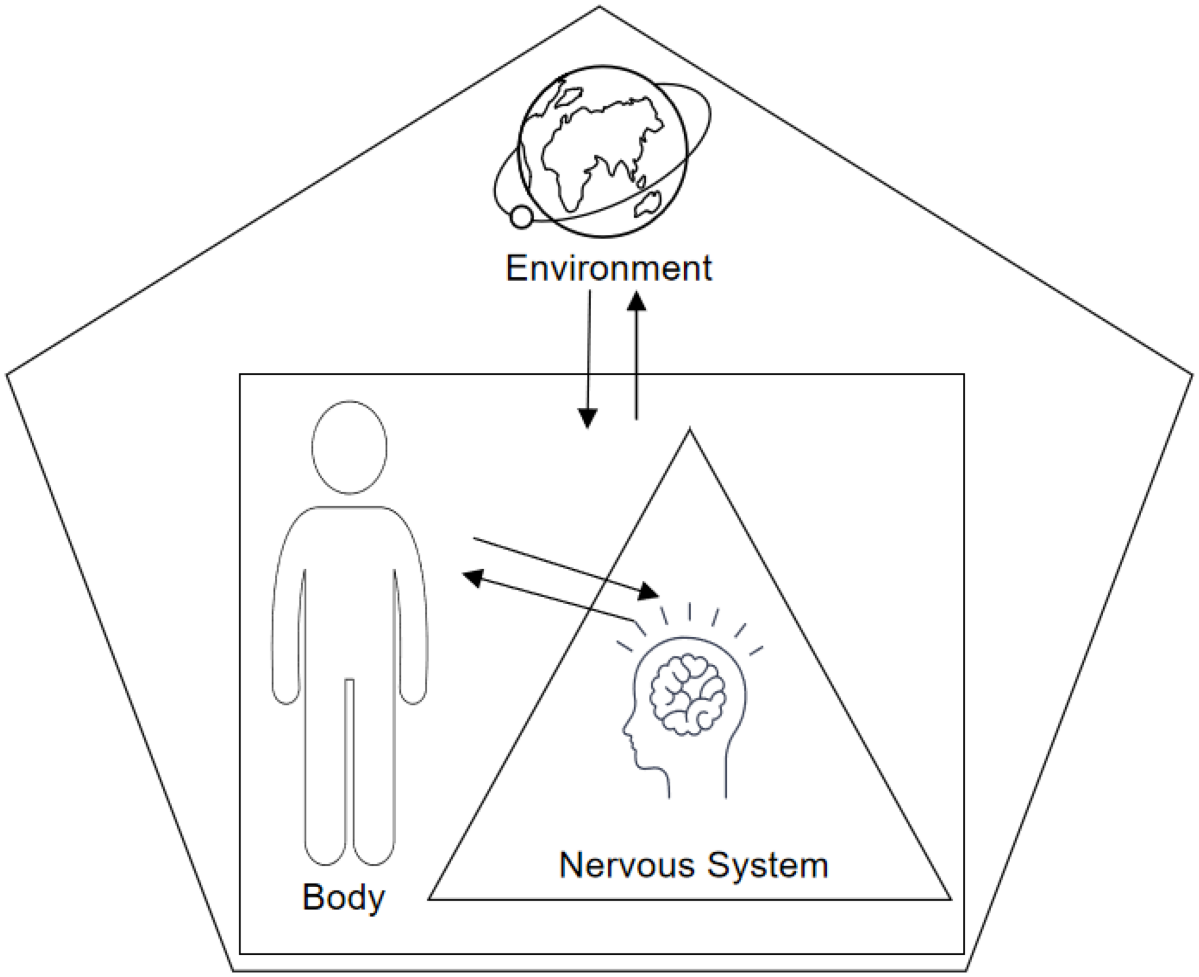
Contextualized body and environment from the perspective of the dynamic system.

It is worth noting that when studying the laws of social psychology, the research object can always be divided into neurons to the brain, and then from reaching the individual, the individual to the group, and the social level. But in actual social psychology research, the needs of social psychology research can be selected and classified at different levels. Figure 5 shows the emotion-based body and environment from a dynamic system perspective.

As shown in Figure 5, it should be noted that in terms of social psychology-based dynamic research systems, the differences between the brain and the body, individuals and communities, etc., follow the laws of the classical scientific research process. But in fact, the two are nested together, dependent on each other but not independent. This article calls this state “separated but not separated.”

### Psychodynamic View of Dynamical System Theory

**Figure 6:**
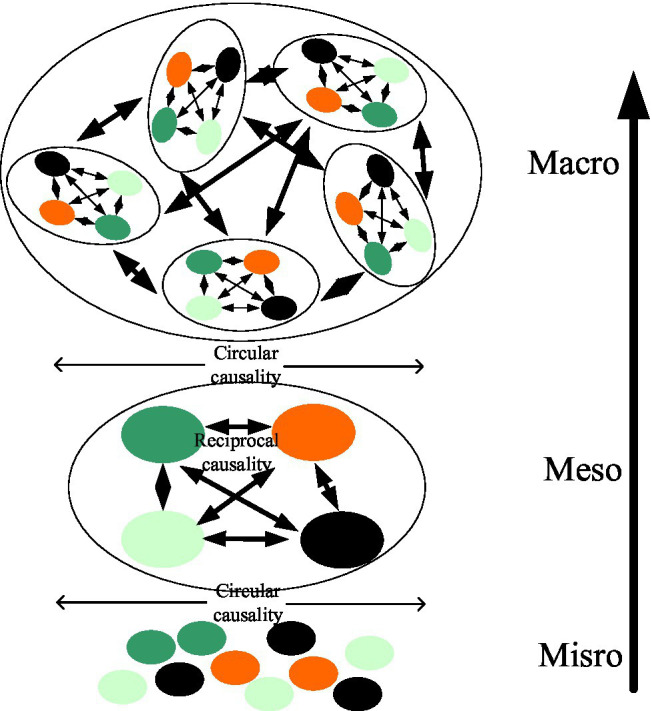
System causality.

Compared with the traditional development model, the dynamic system based on social psychology highlights a causal relationship. In this sense, development can be thought of as a dynamic system of continuous change, in which the components of the system express a relationship of interaction in an unbalanced development. A non-linear relationship would mean a relationship that is inseparable but simply superimposable, where the relationship between elements is more important than the components themselves, so the system must be constrained by the relationships between the elements rather than the components themselves. Essentially, systems can be divided into two types, as shown in Figure 6.

As shown in Figure 6, one is the interaction of components in the same process, called inversion. In this interaction, different elements are related to each other, and it is difficult to distinguish which is the first and which is the last. Others also have many levels of relationships, that is, cause and effect. For example, it uses the personal perspective as a low-level unit of consideration that can include the thoughts, opinions, and emotions of many individuals. And at higher levels of research, individuals exhibit a degree of stability in their global understanding. The second can refer to a dynamic process from the top down, similar to controlling the system as a whole. This result is achieved by selecting or adding activities to some lower level elements. For example, the stability of an individual’s perception of the world affects the emotions and perceptions that are part of it. Under these two interactions, the system continues to evolve into a possibility. In conclusion, a strong sociological dynamical system implies that different role factors operate in a non-linear relationship during the development of an individual’s brain. No environmental or biological factor can be regarded as the final developmental result of psychological research. Furthermore, the effects of these different factors on individual development are not mutually exclusive. Seemingly inadvertently subtle, but likely to lead to a larger impact.

## The Development Orientation of the Concept of Justice in Social Psychology

### Status Quo of Social Psychology’s View of Justice

#### Purpose of the Investigation

At present, China’s economy is booming, but due to the increasingly intensified social contradictions, some social psychological problems are gradually emerging. This paper takes people’s social psychology views of justice as the research object and uses the data of college students’ psychological questionnaires in the cloud databases of major schools of cloud computing to calculate and analyze. And it compares and analyzes pre-existing behavior, thereby making the data collected more and more accurate. Through the research on the current situation of college students, this paper finds the existing social psychological problems, analyzes the possible causes, and then investigates and analyzes the research orientation of people’s social psychology, so as to provide a basis for cultivating college students’ mental health. College students are in a critical period of value formation and are easily affected by the social environment, and some injustices in colleges and universities also affect college students’ view of justice.

#### Investigation Object

This research takes college students from major universities as the research object and uses cloud computing technology to integrate the most representative questionnaires in the databases of major universities. A total of 277 electronic questionnaires were integrated in this study. The questionnaire is mainly designed in three aspects: first, the investigation is carried out from the perspective of college students’ theoretical understanding of the concept of justice. The second is to investigate the degree of recognition and evaluation of college students on the current social justice environment in China. The third is a survey of current college students’ understanding and evaluation of some injustices in colleges and universities. The basic statistical data of the research subjects are shown in Table 1.

**Table 1:** Statistical table of the basic situation of the surveyed individuals.

Sample category		Quantity (person)	Proportion (%)
Gender	Male	152	54.9
	Female	125	45.1
Only child	Yes	130	46.9
	No	147	53.1
Professional category	Literature and history	85	30.7
	Science and engineering	192	69.3
Grade	Freshman	45	12.6
	Sophomore	37	9.7
	Junior year	76	23.8
	Senior year	119	38.6

#### Investigation Content

In this paper, a random sample is collected for a survey of college students’ social psychology issues, and 23 simple and easy-to-understand questions are selected.

### Survey Results of the Status Quo of Social Psychology

#### College Students’ Theoretical Understanding of the Concept of Justice

In order to evaluate the current situation of college students’ understanding of the concept of justice, this paper conducts a calculation and analysis of cloud data on a series of questions about the concept of justice of college students. It can be seen that the students’ understanding of the concept of justice is shown in Table 2.

**Table 2:** Knowledge of the concept of justice data sheet.

	Know a lot (%)	Comparative understanding ratio (%)	General understanding ratio (%)	Little is known about the proportion (%)
Freshman	8.6	28.6	54.3	8.6
Sophomore	7.4	29.6	55.6	7.4
Junior year	15.2	34.8	47.0	3.0
Senior year	9.3	31 8	54.2	4.7
Postgraduate	11.9	40.5	40.5	7.1
Summary	108	33.2	50.5	5.4

As shown in Table 2, 10.8% of the college students choose “a lot of knowledge”; 33 2% of the college students choose “a little understanding”; 50.5% of the college students choose “general understanding”; 5.4% of the college students choose “little understanding.” It can be seen from this that the vast majority of college students understand the concept of the concept of justice, while a small number of students rarely understand the concept of justice.

**Figure 7:**
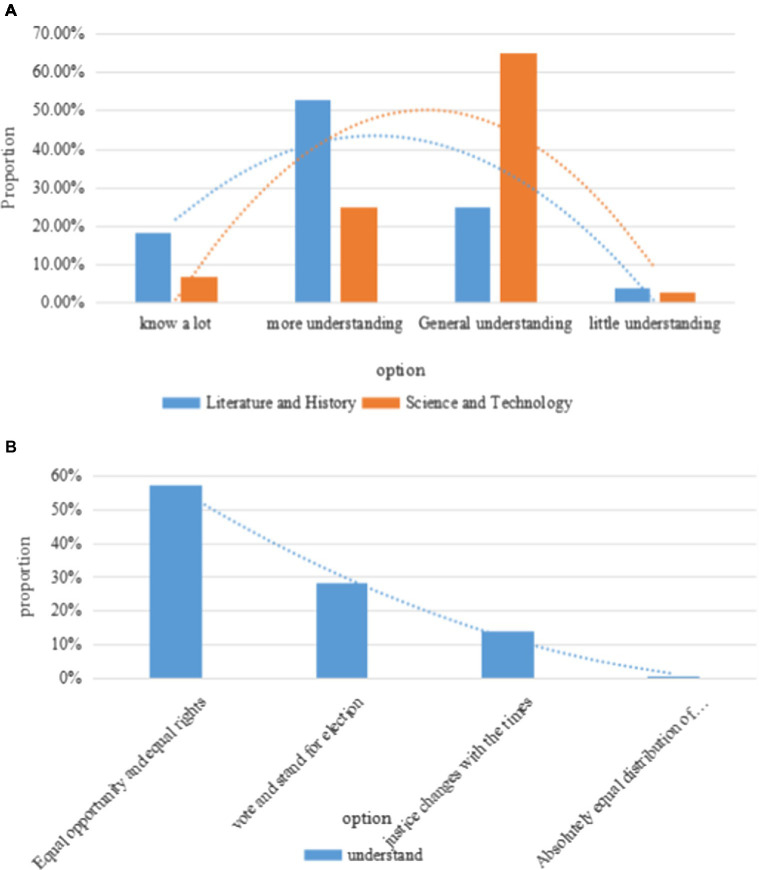
Different majors’ understanding of the concept of justice and its connotation. **(A)** Knowledge of the concept of justice. **(B)** Understanding of the connotation of the concept of justice.

This paper analyzes the understanding of the concept of justice among students of different majors, and the experimental investigation results are shown in Figure 7.

As shown in Figure 7, the study also found that there are significant differences in the cognition of the concept of justice among college students with different professional backgrounds. There are more literature and history students who choose “very concerned” and “relative attention” than science and engineering students; fewer literature and history students choose “general attention” and “almost no attention” than science and engineering students. It can be seen that the students of literature and history are more concerned about social justice issues than the students of science and engineering. This phenomenon may be related to the content of education and the direction of interest. The survey on “understanding the concept of justice” shows that 57% of students choose “everyone should enjoy equal opportunities and equal rights,” and only 0.7% of college students choose “absolutely equal distribution of material resources.” It can be concluded that students have different views on the concept of justice concept due to their own senses and thoughts, but most people have a relatively basic understanding of it, and only 0.7% of students do not understand this theoretical concept. The specific reasons require further analysis of the data. Although college students have different feelings about society and different views on justice issues, they can basically make reasonable judgments. Only 4% of college students think that social justice issues are serious.

#### The Degree of Attention of College Students to Social Justice

The degree of attention indicates the degree of attention to an event or object, and the higher the degree of attention, the higher the degree of attention. This paper investigates the data of college students’ concerns about social justice and integrates it, so as to explore the research orientation of people’s view of justice in social psychology. The experimental results are shown in Table 3.

**Table 3:** Statistical table on the level of concern for social justice.

	Know a lot (%)	Comparative understanding ratio (%)	General understanding ratio (%)	Little is known about the proportion (%)
Freshman	14.3	57.1	25.7	2.9
Sophomore	11.1	51.9	33.3	3.7
Junior year	15.2	47.0	34.8	3.0
Senior year	12.1	51.4	34.6	1.9
Postgraduate	16.7	45.2	35.7	2.4
Summary	13.7	50.2	33.6	2.5

As shown in Table 3, the survey results of college students’ attention to social justice show that 16.7% of college students chose “very concerned,” 50.2% of college students chose “relative attention,” and 33.6% of college students chose “general concern,” only 2.5% of college students chose the “hardly concerned” option. It can be seen that most college students pay more attention to social justice events, and this attitude shows that most of them have a sense of justice to a certain extent.

**Figure 8:**
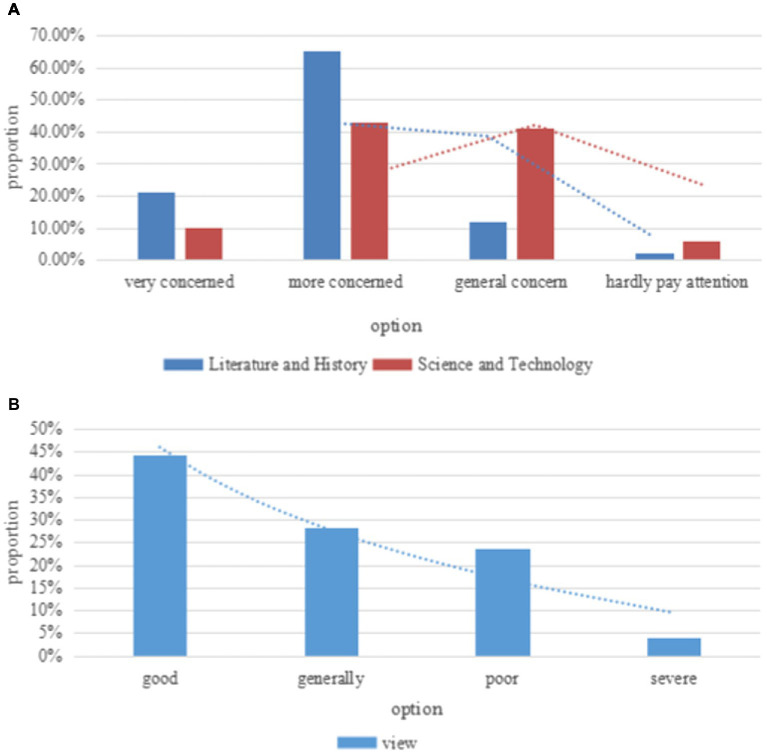
The concerns and opinions of college students with different majors on social justice issues. **(A)** Concerns about social justice issues. **(B)** Views on social justice issues.

Figure 8 shows the concerns and views of college students with different majors on social justice issues.

As shown in Figure 8, 44.4% of college students believe that the current social justice is good, 28.1% of the college students think that the current social justice problem is average, 23.5% of the college students think that the current social justice problem is poor, and 4% of the college students think that the current social justice problem is more serious.

### Problems Existing in the Orientation of College Students’ View of Justice

**Figure 9:**
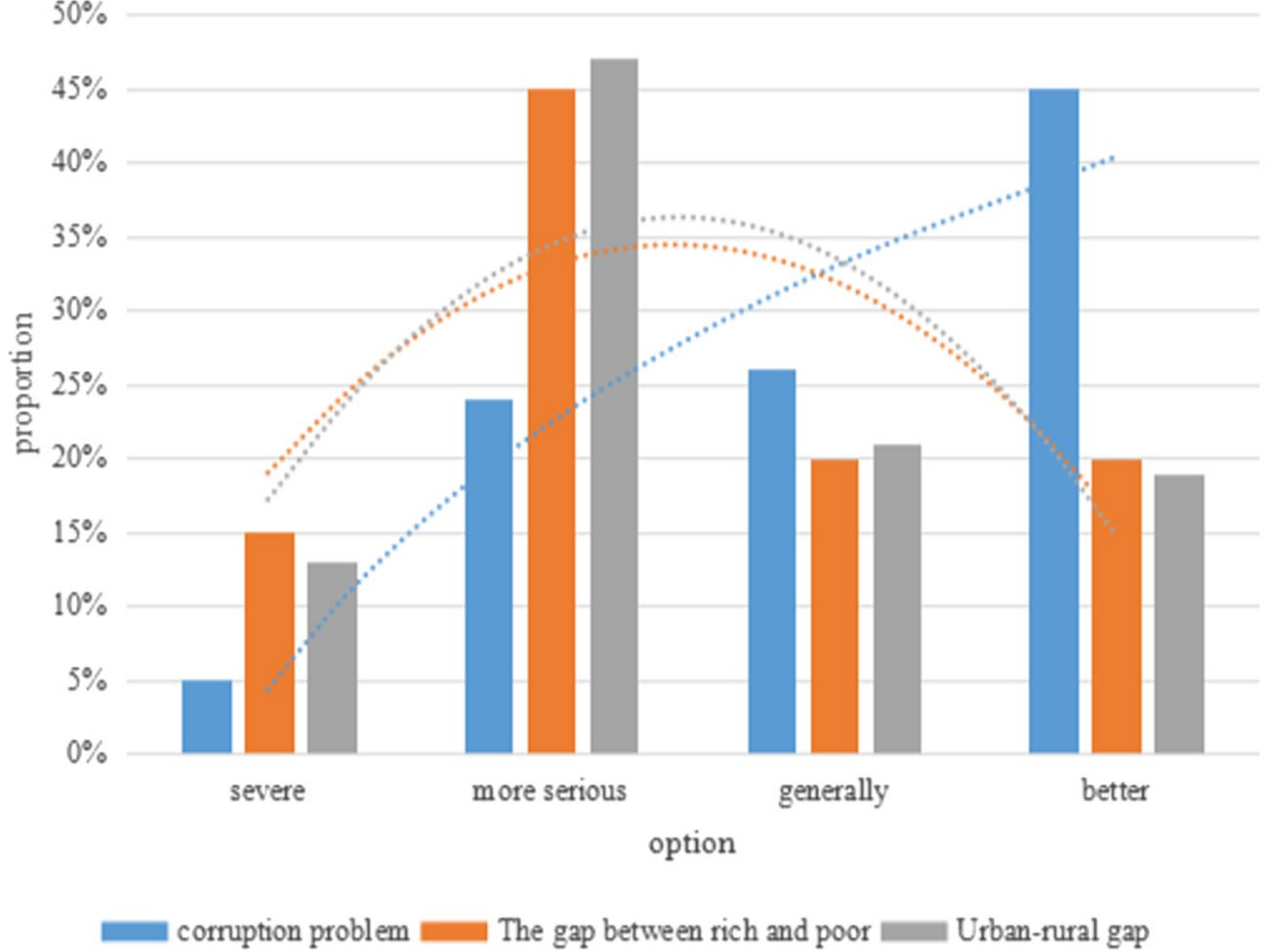
College students’ satisfaction with social justice issues.

At present, in the field of social psychology, students have some misunderstandings in the concept of justice, especially they are dissatisfied with social justice and lack a sense of justice. This paper conducts a survey and analysis on the satisfaction of college students’ social justice issues, and the experimental survey results are shown in Figure 9.

After analysis, it can be concluded that college students are not satisfied with social justice issues, such as corruption, the gap between the rich and the poor, and the gap between urban and rural areas. Among them, the choices on the gap between the rich and the poor and the gap between urban and rural areas are roughly the same.

## Conclusion

The application of cloud computing technology to the research and development of social science practice can enhance the effectiveness of research methods in social psychology. This enables researchers to monitor and record the psychological behavior of individuals exhibited by researchers, and to provide researchers with effective technical support. After the birth of social psychology, many scholars from different fields have conducted research on the topic of social psychology. However, early scientific research is often more “one-sided.” Furthermore, many studies have explored through simple linear structures. Social psychology is a complex science, and social psychology has greatly improved our understanding of the development of individual and group behavior. Strengthening the cultivation of people’s social psychological concepts is an important part of the cultivation of people’s mental health, which is crucial to personal development. At the same time, the concept of justice is also a psychological concept. Therefore, this work chooses to explore people’s attitudes toward justice and the problems from the perspective of social justice, aiming to lay a foundation for the research orientation of social psychology. However, there are still many deficiencies in the research: in the elaboration of justice and social psychology related theories, the theoretical foundation should be deepened; the selection of questionnaire samples is limited to college students in my school, and the survey results have certain limitations.

## Data Availability Statement

The original contributions presented in the study are included in the article/supplementary material, further inquiries can be directed to the corresponding author.

## Author Contributions

This paper was written by YZ, who was responsible for the first draft, and DD was responsible for the statistics of the paper. All authors contributed to the article and approved the submitted version.

## Funding

This work was supported by Humanities and Social Science Project of Shandong Province (project no: 2020-ZXGJ-05) and Special Key Project of China Association of Higher Education (project no: 2019FDYZD01).

## Conflict of Interest

The authors declare that the research was conducted in the absence of any commercial or financial relationships that could be construed as a potential conflict of interest.

## Publisher’s Note

All claims expressed in this article are solely those of the authors and do not necessarily represent those of their affiliated organizations, or those of the publisher, the editors and the reviewers. Any product that may be evaluated in this article, or claim that may be made by its manufacturer, is not guaranteed or endorsed by the publisher.
